# Need for more evidence in the prevention and management of perinatal asphyxia and neonatal encephalopathy in low and middle-income countries: A call for action

**DOI:** 10.1016/j.siny.2021.101271

**Published:** 2021-10

**Authors:** Vaisakh Krishnan, Vijay Kumar, Gabriel Fernando Todeschi Variane, Waldemar A. Carlo, Zulfiqar A. Bhutta, Stéphane Sizonenko, Anne Hansen, Seetha Shankaran, Sudhin Thayyil, Newborn Brain Society Guidelines and Publications Committee

**Affiliations:** a Centre of Perinatal Neuroscience, Department of Brain Sciences, Imperial College London, London, UK; b Protecting Brains & Saving Futures, Brazil; c Division of Neonatology, University of Alabama at Birmingham and Children's Hospital of Alabama, Birmingham, USA; d Centre for Global Child Health, Hospital for Sick Children, Toronto, Canada; e Center of Excellence in Women & Child Health, The Aga Khan University, Karachi, Pakistan; f Geneva University Hospitals, Department of Pediatrics, Switzerland; g Division of Newborn Medicine, Boston Children's Hospital, Boston, USA; h Wayne State University, Detroit, USA

**Keywords:** Newborn infant, Neonatal encephalopathy, Neonate, Hypothermia, Low- and middle-income countries, LMICs, Low- and Middle-income countries, ASM, Anti-seizure medication, HELIX, hypothermia for moderate or severe neonatal encephalopathy in low and middle-income countries trial, HICs, High-income countries, NE, neonatal encephalopathy, WHO, World Health Organization

## Abstract

Although low- and middle-income countries (LMICs) shoulder 90 % of the neonatal encephalopathy (NE) burden, there is very little evidence base for prevention or management of this condition in these settings. A variety of antenatal factors including socio-economic deprivation, undernutrition and sub optimal antenatal and intrapartum care increase the risk of NE, although little is known about the underlying mechanisms. Implementing interventions based on the evidence from high-income countries to LMICs, may cause more harm than benefit as shown by the increased mortality and lack of neuroprotection with cooling therapy in the hypothermia for moderate or severe NE in low and middle-income countries (HELIX) trial. Pooled data from pilot trials suggest that erythropoietin monotherapy reduces death and disability in LMICs, but this needs further evaluation in clinical trials. Careful attention to supportive care, including avoiding hyperoxia, hypocarbia, hypoglycemia, and hyperthermia, are likely to improve outcomes until specific neuroprotective or neurorestorative therapies available.

## 1 Introduction

Of 53 million children aged less than 5 years with developmental disabilities, 50 million live in low and middle-income countries (LMICs) [1]. Almost 80 % of these disabilities are related to a perinatal brain injury, of which neonatal encephalopathy (NE) is the biggest cause. The burden of NE across the globe is massive, with an estimated 1.2 million neonates affected each year and a significant proportion of them dying or left with permanent neurodisability [2]. Again, over 90 % of the disease burden occurs in LMICs (5–20 per 1000 live births), where the incidence is 5–20 times higher than that of high-income countries (HICs) (1–2 per 1000 live births) [3]. The international attention has been primarily focused on neuroprotection in HICs and quite rightly, on community-based setting in LMICs, where most births used to occur. These efforts have led to a major shift from home births to facility-based deliveries, and a reduction in maternal and neonatal mortalities, particularly in South Asian countries [4].

However, NE following hospital births in LMICs still remains unchanged in the past decade and 10 to 20 times higher than that of HICs, and now the focus needs to change from ‘survival’ to ‘intact survival’. Very little evidence is available on prevention and management of NE in LMICs hospital settings. Consequently, much of our current understanding about NE in LMICs is based on extrapolations from studies in HICs and speculations without adequate local evidence base.

The recent data from the world largest cooling trial (HELIX: Hypothermia for moderate or severe Neonatal Encephalopathy in Low and Middle-income countries) clearly demonstrating harms of therapeutic hypothermia in South Asian tertiary neonatal intensive care units [5], not only highlights the dangers of such an approach, but also challenges some of the long-held assumptions about the origins of brain injury in NE. NE is one of the strongest examples of the ‘Inverse Care Law’ in research; medical care [quantity and quality of research] is inversely proportional to the needs of the population. Finally, the HELIX trial prompts us to ask difficult questions about ethnic, socio-economic determinants of health, intrinsic population differences and in-equalities that exist even in HICs, and how that might influence outcome of neonates, even when state-of-art care is available.

In this review, we critically examine the evidence behind some of the commonly used preventive and management strategies in NE, highlight key knowledge gaps, provide suggestions for high-quality NE research in LMICs.

### 1.1 Antenatal factors in NE

Proximal health determinants like socio-economic deprivation, maternal illiteracy and unemployment, maternal co-morbidities including hypertension and poor nutrition, and distal health determinants such as socio-cultural factors, child marriages, high home delivery rates, health system factors and failures are all associated with increased incidence of birth asphyxia in LMICs [6].

A systematic review and meta-analysis of 12 studies from Sub Saharan African countries reported that even one antenatal assessment by a skilled person reduced neonatal mortality (Relative risk (RR) 0.61; 95 % Confidence Interval (CI), 0.43–0.86); a significant proportion of these deaths may be due to NE [7]. A case control study involving 88 neonates with NE and 176 control neonates in Ethiopia reported maternal illiteracy (adjusted odds ratio (AOR) 6; 95 % CI, 1.51–23.8), primiparity (AOR 3.1; 95 % CI, 1.51–6.38), antepartum hemorrhage (AOR 12; 95 % CI, 2.29–63.11) and meconium stained amniotic fluid (AOR 7.88; 95 % CI, 2.92–21.29) as independent risk factors for birth asphyxia [8]. In another prospective cohort study of 248 term and near-term emergency obstetric referrals in New Delhi, Rani et al. reported low socio-economic status, inadequate antenatal care and poor intrapartum care increased the risk of intrapartum death [9]. In a cohort study involving 3189 mothers, Lee et al. reported a synergistic risk of maternal-fetal disproportion and birth asphyxia. The risk of asphyxia for 3.3 kg neonates born to a mother shorter than 145 cm was 3.8 (95 % CI, 2.2–6.5) higher than a 2.6 kg neonate born to a mother taller than 145 cm in this study [10].

An unmatched case control study from Uganda involving 210 term neonates reported that antenatal factors such as hypertension in pregnancy in increases the risk of NE (AOR 3.8; 95 % CI, 1.49–9.5) [11]. Another study from Ghana reported a high incidence of birth asphyxia in neonates born to mothers who did not receive nutritional counseling during pregnancy (AOR 5.6; 95 % CI, 1.5–21.6).[12]

Nevertheless, very little is known about the mechanisms by which these antenatal factors increase the risk of NE.

### 1.2 Intrapartum factors in NE

Although anecdotally, NE is often attributed to an intrapartum event, a causal association is difficult to prove in the absence of an acute perinatal sentinel event. In a prospective study from Nepal, Ellis et al. reported intrapartum sentinel events (obstructed labor, cord prolapse, uterine rupture, antepartum bleed, eclampsia) in 28 (22 %) of 131 neonates with NE as opposed to 17 (3 %) of 635 control neonates [13]. Cooling trials in LMICs have reported a much lower incidence of perinatal sentinel events. Bharadwaj et al. reported perinatal sentinel events in 1.6 % of the cooled neonates [14]; Joy et al. reported perinatal sentinel events in 3.5 %; Gane et al. reported perinatal sentinel events only in 2 % of the neonates with NE [15,16]. The HELIX trial reported perinatal sentinel events in 13 % of neonates with NE [5].

A recent systematic review involving a total of 23,383 term deliveries reported an increased incidence of NE (Odd ratio (OR) 2.25: 95 % CI, 1.63–3.12) with intrapartum oxytocin use [17]. Given that induction and augmentation of labor with oxytocin without the use of infusion pumps is a common practice in LMICs, this is an area that requires further research.

### 1.3 Co-existent perinatal sepsis, chorioamnionitis and NE

Contrary to popular belief, there are no data to suggest that the incidence of co-existent blood stream positive sepsis and NE in LMICs is higher than that of HICs. Even by using a combination of blood cultures and polymerase chain reaction in a Sub Sharan neonatal unit, co-existent sepsis could be identified in less than 9 % of the neonates with NE [18]; a very similar incidence to that of HICs [19]. Cooling trials in LMICs have reported co-existent blood stream positive infection in 5 %–10 % (Table 1), while HICs have reported blood stream positive infection in 5 %–17 % of neonates with NE [19,20]. In the HELIX trial, co-existent sepsis was unrelated to lack of hypothermic neuroprotection [5]. While group B streptococcus is the predominant organism in HICs, gram negative bacteria are far more common in LMICs [19], and group B streptococcus is uncommon [5].

**Table 1:** Summary of the 14 pilot trials and two clinical trials (phase III)* of therapeutic hypothermia from low and middle-income countries [[66], [67]].

Author, Year	Country	Cooling method	Number: cooled/control	Primary Outcome	Mortality: cooled/control	Neurodisability at 18 months or more	Result
Akisu, 2003	Turkey	Cooling head cap	11/10	Abnormal EEG and reduction in CSF platelet activating factor (PAF) levels.	0/2	Not available	Only significant reduction found in abnormal EEG. Reduction in CSF – PAF in cooled babies.
Bhat, 2006	India	Unknown device	20/15	Death/abnormal neuro exam at discharge	3/5	Not available	No difference in mortality, lesser abnormal neuro exam in cooled group
Lin, 2006	China	Cooling head cap	32/30	Neonatal behavioural neuro assessment (NBNA) at 7–10 days/Brain injury on CT scan	2/2	Not available	Improvement in injury on CT scans and NBNA
Robertson, 2008	Uganda	Water bottles	21/15	Death/seizures/abnormal neuro exam	7/1	Not available	Higher mortality in cooled group
Zhou, 2010*	China	Cooling head cap	100/94	Death/severe disability at 18 months	20/27	Yes	Lesser death/severe disability in cooled group
Bharadwaj, 2012	India	Gel packs	62/62	Death/Developmental delay at 6 months	3/6	Not available	No difference in mortality
Joy, 2012	India	Gel packs	58/58	Oxidative stress	1/4	Not available	Lesser oxidative stress in cooled group
El Shimi, 2013	Egypt	Gel packs	10/10	Brain-derived neurotrophic factor (BDNF) and Neuron Specific Enolase (NSE), MRI brain	4/8	Not available.	Mortality, MRI score lower in cooled group.
Gane, 2013	India	Gel packs	60/60	8-hydroxy2-deoxyguanosine levels	4/8	Not available.	Lower 8-hydroxy2-deoxyguanosine levels in cooled group
Thayyil, 2013	India	Phase change material	17/16	MRI imaging biomarkers	4/2	Not available	Higher mortality in cooled; no reduction in whole brain fractional anisotropy on tract based spatial statistics with cooling
Thanigasalam, 2015	India	Gel packs	60/60	Acute kidney injury	16/30	Not available	Cooling reduced acute kidney injury
Rakesh K, 2017	India	Phase change material	60/60	Myocardial dysfunction	9/16	Not available	Reduced myocardial dysfunction in cooled group
Chen, 2018	China	Cooling head cap	18/18	Death/Severe disability at 15 months	0/1	Not available	No difference in primary outcome
Aker 2019 (THIN study)	India	Phase change material	11/11	Magnetic resonance imaging (MRI)	2/1	Not available	Seriously underpowered study to make any valid scientific conclusions.
Catherine, 2020	India	Phase change material	76/79	Death/Neurodisability at discharge and at 18 months of age	22/29	Yes	No difference in primary outcome
Thayyil, 2021 (HELIX trial)*	India, Sri Lanka, Bangladesh	Tecotherm Neo	202/206	Death/Neurodisability at discharge and at 18 months of age	84/63	Yes	No difference in primary outcome; significant increase in mortality

It is important not to confuse neonatal sepsis with chorioamnionitis and these conditions do not necessarily overlap. Chorioamnionitis is reported in approximately 4 % of the healthy term neonates and in 11–20 % of neonates with NE and increases the risk of NE (OR 3.5; 95 % CI, 2.1–5.8)[21]. In a cohort of 50 neonates with NE undergoing cooling therapy in the USA, Johnson et al. reported clinical chorioamnionitis in 10 (20 %), histologic chorioamnionitis in 16 (32 %), histologic funisitis in 10 (20 %) and neonatal bacteremia in 4 (8 %) neonates. In another cohort of 210 neonates with NE (60 [29 %] had placental examination) admitted to the neonatal unit in Uganda, Tann et al. reported histological chorioamnionitis without funisitis in 10/60 (16.7 %), funisitis in 16/60 (26.7 %), and bacteremia in 18/210 (8.6 %) [22]. In this study, only funisitis was associated with NE and chorioamnionitis without funisitis was not [22]. In the HELIX trial, funisitis was seen in 17 % of the neonates [5]. The association of fetal inflammation and NE requires further careful exploration.

### 1.4 Interventions for preventing NE

#### 1.4.1 Institutionalized deliveries

Although the efforts in LMICs have been rightly focused on reduction of maternal and neonatal mortality by increasing institutionalized deliveries, these may have a secondary effect on reduction in NE. Mathematical modeling by Lee et al. on data from 184 countries over 20 years from 1990 to 2010 reported that incidence of NE has decreased from 11.7 to 8.5 per 1000 live births, due to increased institutionalized deliveries and better intrapartum care [2].

India's conditional cash transfer program where cash incentives are provided to families for giving birth in a health facility has significantly increased the proportion of institutionalized deliveries and reduced perinatal mortality (i.e. still birth after 28 weeks or death of a baby within 7 days of birth) [23]. Over 90 % of deliveries now occur in health care facilities in India, and these figures go up to 99 % in South India. A prospective study involving 18 large tertiary hospitals in India reported a NE (grade non-specified) incidence of 14 per 1000 live births, amongst inborn neonates in 2003 [24]. The emerging data from an ongoing large prospective study in India in three large tertiary hospitals in South India (PREVENT study: ClinicalTrials.gov: NCT04054453) report an incidence of moderate or severe NE in 14 per 1000 live births amongst inborn neonates, in 2020.

While prompt access to emergency caesarean section is vital, excessive caesarean sections without a concomitant reduction in NE have become a major health care problem, particularly in private health care settings in India [25].

#### 1.4.2 Labor support

Emotional support (e.g., reassurance and praise), comforting measures (e.g., touch, massage, warm baths/showers, promoting fluid intake), encouragement and provision of information (e.g., coping methods, update on progress of labor) provided by birth companions improves the labor experience and reduces birth asphyxia (low Apgar scores). The Cochrane meta-analysis of 26 studies, of which 13 were from HICs and 13 were from LMICs, showed labor support by birth companions reduces the need for a caesarean section (RR 0.75; 95 % CI, 0.64–0.88) or instrumental delivery (RR 0.90; 95 % CI, 0.85–0.96), and the chances of having a neonate with a low five‐minute Apgar score (RR 0.62; 95%CI, 0.46–0.85) [26]. In another systematic review, a continuous one-to-one support during labor reduced the number of neonates with low 5-min Apgar score (13 trials, n = 12,515, RR 0.69; 95 % CI, 0.50–0.95) [27]. Although these studies have been underpowered to examine a reduction in NE, the benefits on maternal outcomes are convincing. While the presence of birth companions, particularly male partners during delivery is the standard practice in HICs, this is not often the case in LMICs, particularly in Africa and South Asia where considerable cultural and logistic barriers still exist.

#### 1.4.3 Fetal surveillance

Continuous electronic fetal monitoring using cardiotocography in a Cochrane review was found to be cost ineffective and increased the rates of interventions (10 trials, n = 18,615, RR 1.15; 95 % CI, 1.01–1.33, low quality evidence) and caesarean sections (11 trials, n = 18,861, RR 1.63; 95 % CI, 1.29–2.07, low quality evidence) in HICs [28]. Cardiotocography in comparison to intermittent auscultation showed no significant improvement in overall perinatal death rate (11 trials, n = 33,513, RR 0.86; 95%CI, 0.59–1.23, low quality evidence), but reduced the neonatal seizure rates (9 trials, n = 32,386, RR 0.50; 95%CI, 0.31–0.80, moderate quality evidence) [28].

A systematic review by Blix et al. showed better detection of abnormal fetal heart rate using a Doppler device than a Pinard stethoscope (RR 1.77; 95%CI, 1.29–2.43) [29]. However, these devices provide only a cross sectional information on the fetal heart rate and may not detect a compromised fetus till there is a sudden fetal heart disappearance or a severe bradycardia. An alternative approach is checking for heart rate changes with fetal movements and speed of recovery of the fetal heart rate after each contraction, using a graphical display doppler. This concept of *“intelligent intermittent auscultation”,* provides reassurance of the fetal wellbeing and may detect a compromised fetus much earlier. Graphic display dopplers could be a promising tool for surveillance in the low-resource settings, but the effectiveness of such monitoring needs to be evaluated in future trials.

#### 1.4.4 Partogram

Documenting the labor progress, particularly cervical dilation, uterine contractions and descend of the fetal head (i.e., partogram) is important not just for medical record keeping but for making real time decisions in case the labor progress deviates from the accepted norms. The partograms have been in clinical use for more than half a century since the original description of the Friedman curves [30]. This study published in 1955, included 500 women (55 % forceps delivery), many of whom were heavily sedated during labor, and reported average labor progression (cervical dilatation in active stage) as approximately 1 cm per hour. This study formed the basis of action and alert lines in the partogram, when the progress was sub-optimal, and to undertake prompt interventions [30].

While the partogram has been modified several times, is consistently recommended by the World health Organization (WHO), the evidence base for this tool is weak. A Cochrane review including 11 studies on use of paper partogram involving 9475 women performed found no significant reduction in caesarean rates (3 trials, n = 1813, RR 0.77; 95%CI, 0.40–1.46; I^2^ = 87 %; very low‐quality evidence) or low Apgar scores (<7 at 5 min) (2 trials, n = 1596, RR 0.76; 95 % CI, 0.29–2.03; I^2^ = 87 %; very low‐quality evidence). A realistic review of the partogram by the same authors, looked more deeply into the reasons why partogram did not work as it was intended to [31]. One key factor is that the partograms are often filled in retrospectively or are ignored in resource-limited settings, usually due to the intense workload and staff shortage.

The feasibility of electronic partogram (e-partogram) to ensure real-time recording has been studied in various LMICs [32]. A prospective study from Bangladesh involving 5230 deliveries comparing paper partograph versus the e-partogram reported reduced caesarean section rates (36 %–25 %, p = 0.001) and prolonged labor with the use of e-partogram [33]. The user rate of e-partogram showed three-fold increase (OR 3.31; 95 % CI, 2.04–5.38; p < 0.001) compared to the user rate with paper partogram. Similarly, a study from Kenya comparing 842 women monitored with e-partogram and 1042 women with paper partogram showed usage of e-partogram associated with a 56 % (95%CI, 27 %–73 %) lower likelihood of adverse fetal outcomes as compared to monitoring with paper partogram [34].

A recent prospective study in Nigeria and Uganda conducted by the WHO involving 9995 women reported that the labor progression was extremely variable, and the rate of cervical dilatation had no effect on the fetal outcomes [35]. Following this study, the WHO renamed partogram as ‘labor care guide’ and removed the alert and action lines to allow individualization of the labor progress [36]. While this has been widely implemented by the WHO, it is important to note that the effect of the WHO labor care guide on NE has not been evaluated yet, and the recommendations about labor progression are solely based on the data from Africa [36]. It is unclear if these data are applicable to countries outside Africa, and whether allowing for longer time in active labor would increase NE. Multi-country clinical trials of the new WHO labor care guide are currently ongoing.

### 1.5 Neonatal interventions

#### 1.5.1 Neonatal resuscitation

Of the 136 million neonates born each year, 5–10 % fail to initiate spontaneous breathing [37]. Although the majority require only gentle stimulation, 3–6% require basic neonatal resuscitation and approximately 1 % may need more advanced neonatal resuscitation.

The First Breath study group investigators (Helping Babies Breathe) assessed the efficacy of a WHO Essential Newborn Care course (which focuses on routine neonatal care, resuscitation, thermoregulation, breast-feeding, “kangaroo” [skin-to-skin] care, care of the small neonate, and common illnesses) using a before and after study design in 57,643 neonates from rural communities in six countries (Argentina, Democratic Republic of Congo, Guatemala, India, Pakistan, and Zambia) [38]. After birth attendants were trained in the Essential Newborn Care course, the investigators did not find a significant reduction from baseline in the rate of neonatal death in the primary outcome of death within 7 days after birth (relative risk with training, 0.99; 95 % CI, 0.81–1.22) or in the rate of perinatal death, although a significant reduction in the rate of stillbirth (relative risk with training, 0.69; 95 % CI, 0.54–0.88; P = 0.003) was noted [38].

In five of these countries (except Argentina), the investigators also evaluated the effectiveness of a modified version of the American Academy of Pediatrics-Neonatal Resuscitation Program (which teaches basic resuscitation in depth) delivered by birth companions in a cluster-randomized, controlled trial involving 62,366 neonates. No reduction in early neonatal death, stillbirth, or perinatal death was noted in clusters where attendants received training in the Neonatal Resuscitation Program, when compared with the control clusters [38]. It is possible that variations in the rates of facility-based births, and nature of attending personnel might have influenced the trial results i.e. less effective in situations with high facility based births and physician attendance [39]. Subsequent scale up of the program and pre-post evaluation studies has shown a reduction in neonatal mortality and fresh still births in settings with high home birth rates [40].

A meta-analysis involving 2164 term and near-term neonates from 10 randomized and quasi-randomized controlled trials reported a reduced neonatal mortality (RR 0.73; 95 % CI, 0.57–0.94) with room air resuscitation; however, there was no reduction in NE (RR 0.89; 95%CI, 0.68–1.18) or neurodevelopmental impairment at 18–22 months (RR 1.41; 95%CI, 0.77–2.60) [41,42]. These trials were primarily conducted in LMICs more than a decade ago and prior to regular use of oxygen saturation monitoring in labor rooms and comparing the two extremes – room air or 100 % Oxygen. It is unclear if an intermediate concentration (e.g., 30 % Oxygen) would be beneficial for resuscitation in term neonates.

#### 1.5.2 Delayed cord clamping for term neonates requiring resuscitation at birth

A Cochrane review involving 15 trials (from HICs and LMICs) involving 3911 term neonates reported no significant differences between early and late cord clamping for neonatal mortality, Apgar score less than seven at 5 min, or admission to the special care nursery or neonatal intensive care unit. However, mean birth weight was significantly higher in the delayed cord clamping group, when compared with early cord clamping group (101 g increase, 95 % CI 45–157) [43]. Although the improvement in mean hemoglobin levels were transient, neonates in the early cord clamping group were over twice as likely to be iron deficient at three to six months compared with neonates whose cord clamping was delayed (RR 2.65; 95 % CI 1.04–6.73). Use of phototherapy for jaundice was lower in the early clamping group than in the late cord clamping group (RR 0.62; 95 % CI 0.41–0.96). There was no difference in post-partum hemorrhage and use of uterotonic drugs in early and delayed clamping groups. These data suggest modest benefits of delayed cord clamping in term neonates who do not require resuscitation at birth, and this can be easily performed by keeping the neonate over mothers abdomen [43].

There are no safety or efficacy data on delayed cord clamping in term neonates who require resuscitation at birth, either from HICs or from LMICs. It could be argued that if the neonate has no cardiac output, little can be gained by delaying cord clamping, and attempting extensive resuscitation with an intact umbilical cord may not only compromise the resuscitation quality but increase parental stress. Several resuscitators that enable resuscitation with intact cord has been developed [44], but these should not be commercialized in LMICs until safety and efficacy data are available.

#### 1.5.3 Supportive care

Limited data are available on supportive care in NE in LMICs to make definitive recommendations. Almost all interventions used in neonatal units in LMICs are based on evidence from HICs which overlooks population differences, and at times these could have disastrous consequences. In high income country cooling trials, early hypocarbia was associated with adverse outcome at 18–22 months [OR 2.0; 95%CI, 1.1–3.4)][45,46]. Nadeem et al. reported moderate hypocarbia in 69 % of neonates with 10.13039/100006147NE on ventilator support compared to the 31 % not on respiratory support [47]. Klinger et al. reported that severe hyperoxia (pO_2_ > 200 mm of Hg) during the first 2 h after birth was independently associated with adverse outcome. Neonates with a combination of hyperoxia and hypocapnia (<20 mmHg) were more likely to be have a poor outcome at 18–20 months of age (OR 3.07; 95%CI, 1.31–7.18; p = 0.001) [48].

The insensible fluid losses, access used for infusions, ambient temperature and humidity of neonatal intensive care units in LMICs and HICs are likely to be different, which may have an impact on the fluid requirements. Again, limited data are available on optimal maintenance fluid volumes in NE in LMICs, however extreme fluid restriction and fluid overload are both likely to be harmful. Hypoglycemia and hyperglycemia are common in NE and has been associated with adverse outcomes in HICs, although a direct causal effect is unclear [49]. Given the high incidence of growth restriction and subacute brain injury the implications of hypoglycemia in LMICs may be very different to HICs, and optimal thresholds and management strategies may be different. High incidence of epilepsy in LMICs is often attributed to neonatal hypoglycemia, although well-designed prospective studies are limited. Low calcium and magnesium levels are commonly documented in neonates with NE, and so is metabolic acidosis. Although these are routinely corrected, the optimal management strategies for these metabolic perturbations are unclear, due to lack of adequate studies from these settings, particularly on use of sodium bicarbonate for metabolic acidosis.

The supportive care for neonatal units that do not have basic neonatal care as in sub-Saharan Africa [50] would be very different to the situation in south Asian neonatal units with good neonatal intensive facilities, and no generalizations should be made. If a hospital has no facility for basic neonatal care, it would be prudent to establish this first before introducing novel neuroprotective interventions [51].

#### 1.5.4 Coagulopathy and thrombocytopenia

Liver dysfunction with clotting abnormalities and thrombocytopenia are reported in 30 %–50 % of neonates with NE both in HICs and LMICs, although liver dysfunction and visceral bleeding (gastric and pulmonary) appears to be more common in LMICs [52]. Subgaleal bleeds appear to be more common in LMICs and could be life threatening. Hence, it would be prudent to monitor for thrombocytopenia and coagulopathy and correct these derangements promptly. The liver dysfunction may be related to growth restriction and poor maternal nutritional status in LMICs [5]. Again, optimal thresholds for interventions, except for routine use of Vitamin K is unclear.

#### 1.5.5 Neonatal seizures

In HICs, the majority of neonates with moderate or severe NE develop seizures within 24 h of birth, often coinciding with the onset of secondary energy failure. The seizures tend to peak by 36 h before decreasing, and at least 50 % of these seizures are electrical alone, and will be detected only if continuous electroencephalography (EEG) is available [53].

Limited data are available from LMICs on neonatal seizures. In the HELIX trial, the clinical seizure onset appeared to be much earlier than in HICs and many neonates had seizures within few hours of birth [5]. As EEG was not performed in this trial, these need to be confirmed in future studies using continuous EEG monitoring. Lip smacking and other subtle movements can be easily misdiagnosed as seizures and subclinical seizures cannot be detected in the absence of EEG.

Protecting Brains and Saving Futures (PBSF) has developed an advanced telemedicine system to support clinical decisions and provide neurocritical care in Brazil. In this program, experienced remote readers monitor neonates with NE from over 20 different hospitals across the country with aEEG/EEG. Communication between the local equipment and the central server is sent with encrypted data, protecting the privacy of transmitted information. Both access to the monitoring system, management of backup and security services are provided through authentication mechanisms. The feasibility and cost effectiveness of wider implementation of such systems in LMICs remains to be seen.

Little progress has occurred in anti-seizure medication (ASM) therapy in the past decade and the most commonly used first line drug remains phenobarbital. Levetiracetam usage has increased recently given its wide safety margin, availability of pharmacokinetic data and reported efficacy in infants and children. However, a recent ASM trial in HICs reported a higher seizure cessation rate at 24 h (80 % versus 28 %) and 48 h (64 % versus 17 %) with phenobarbital when compared with Levetiracetam. The phenobarbital group had more adverse events, including respiration depression and hypotension [54]. With the currently available evidence, phenobarbital is recommended as the first-line therapy for neonatal seizures. Although administration of ASM is often associated with adverse outcomes in uncontrolled studies, it is difficult to account for the confounding effect of the underlying severity of disease. Considerable knowledge gaps remain in LMIC, both in terms of initiation and discontinuation of ASM and such studies are urgently needed.

#### 1.5.6 Therapeutic hypothermia

A total of 15 pilot randomized controlled trials have been reported from LMICs to date (Table 1). All trials in LMICs, except one [50], were conducted in tertiary neonatal units with good neonatal intensive care and facilities for ventilatory support. A pilot randomized controlled trial of cooling therapy using water bottles in a Sub-Saharan hospital lacking basic neonatal care facilities showed six times higher mortality in the cooled neonates. This was not surprising as cooling therapy should not be administered without adequate supportive care and facilities to monitor oxygen saturation and blood pressure. Although the investigators initially attributed the increased mortality to sepsis rather than lack of supportive care, subsequent studies from the same population reported sepsis was seen in less than 9 % of the neonates with NE [18].

**Fig. 1:**
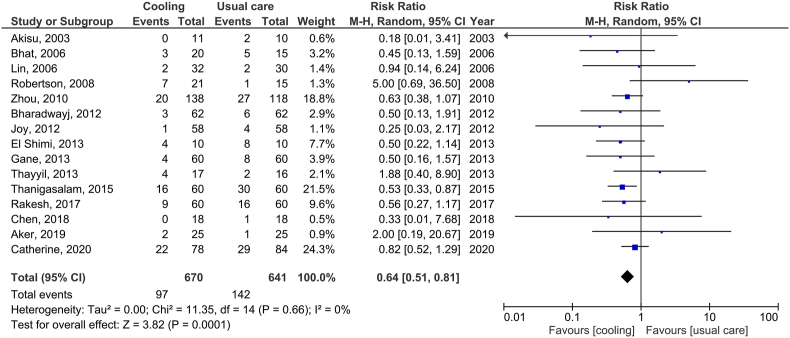
Pooled data from randomized controlled trials of therapeutic hypothermia for neonatal encephalopathy from low and middle-income countries and neonatal mortality, prior to the HELIX trial. Given the sub-optimal quality of the individual studies and possible duplicate publications, it is unlikely that these pooled data are meaningful, and hence are not combined with the HELIX trial data [62,63].

**Fig. 2:**
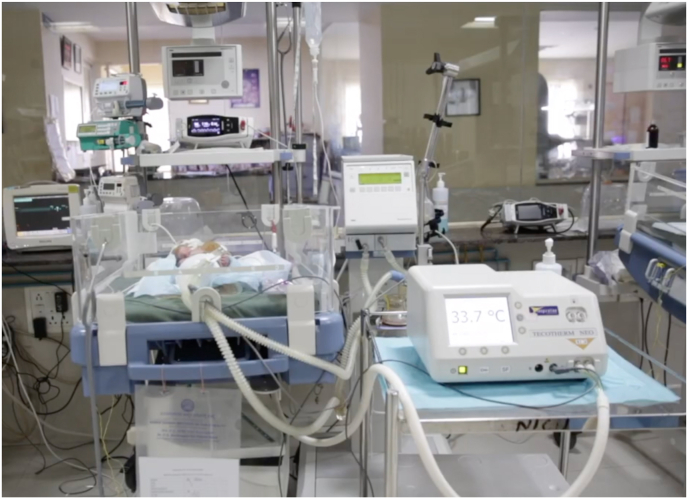
An infant receiving therapeutic hypothermia in a tertiary neonatal intensive care unit at Indira Gandhi Institute of Child Health in Bangalore, India, as a part of the HELIX trial.

The other 14 cooling trials in LMICs were reported from secondary and tertiary neonatal units in India (9 studies), China (3 studies), Turkey (1 study) and Egypt (1 study) (Fig. 2). The largest of these was a clinical trial from China involving 194 neonates [55], but this trial has been heavily criticized for protocol violations, post-randomisation exclusion and poor follow-up rates. Six of these cooling trials were from the same hospital, but with mortality in the usual care arm varying between 7 % and 50 %, raising concerns about data credibility and duplicate publications [[56], [57], [58], [59], [60], [61]]. Hence, although the pooled data from these trials show a reduction in mortality with cooling, such a meta-analysis has no scientific value [62,63] (Fig. 1).

Nevertheless, a number of recent surveys from low-resource settings have reported wide use of therapeutic hypothermia in these settings using a variety of low-cost cooling devices and marked deviations from established cooling protocols in HICs [64,65].

The HELIX trial is the largest cooling trial in the world and the only adequately powered and rigorously conducted clinical trial in LMICs, intended to provide a definite answer to the safety and efficacy of cooling. In the HELIX trial, therapeutic hypothermia was administered using a servo-controlled and automated cooling device (Tecotherm Neo, UK). The trial was conducted in tertiary neonatal intensive care units with excellent facilities for invasive ventilation and cardiovascular monitoring, and access to 3Tesla Magnetic resonance imaging, spectroscopy, and neurodevelopmental follow-up (Fig. 2). Of the 408 neonates randomized to intensive care alone or therapeutic hypothermia with intensive care, the primary outcome (composite of death or disability at 18–22 months) was available in 394 (97 %) of the neonates and occurred in 98 (50.3 %) of the hypothermia and 94 (47.2 %) of the usual care group (RR 1.06; 95%CI, 0.87–1.30); p = 0.55). Eighty-four neonates (42.4 %) in the hypothermia group and 63 (31.3 %) (p = 0.02) neonates in the usual care group died, of whom 72 (35.6 %) and 49 (23.8 %) (p = 0.009) died during the neonatal hospitalization. The need for invasive ventilation (122 (60.4 %) versus 106 (51.5 %); p = 0·069) and inotropic support (161 (79·7 %) versus 128 (62·1 %); p < 0·001) was higher in the hypothermia group. The incidence of both early onset and late onset sepsis was low (less than 10 %) and similar to that reported from HICs, and did not affect hypothermic neuroprotection [5]. Severe microcephaly (17.3 % versus 17 %) and survival without disability (42.3 % versus 34.6 %) were not different between the hypothermic and control groups. The results of the trial were unexpected and disappointing but underline the importance of adequately powered high quality randomized controlled trials before wider implementation of a new intervention [66,67].

Unlike in cooling trials in HICs, very few neonates in the control arm of the HELIX trial had hyperthermia (core temperature >38 °C), but all those who had, either died or had moderate or severe disability. Avoiding accidental hyperthermia is not a trivial task, and lower target set temperatures (e.g. 36 °C) in servo controlled radiant warmers may have to be used. Equally, in low resource sub-Saharan settings without radiant warmers, hypothermia is common, and it is important keep these neonates normothermic.

#### 1.5.7 Generalization of HELIX trial data to other LMICs settings and manual cooling devices

Although the HELIX trial was performed in selected well-resourced tertiary neonatal care units with facilities for invasive ventilation and cardiovascular support, the results are likely to be widely generalizable to all LMICs. A therapy that is unsafe and ineffective alongside optimal intensive care, cannot be safe in less resourced sub-Saharan settings.

In the HELIX trial, an expensive automated servo-controlled cooling device was used for administering therapeutic hypothermia, and hence target temperature was maintained very close to 33.5 °C throughout the cooling period, followed by controlled re-warming at 0.5 °C. Again, if cooling using the best device is harmful, it is unlikely that low technology manual cooling devices would be beneficial. Such devices produce more temperature fluctuations and hence are not approved for clinical use in HICs [68]. Given the harms of therapeutic hypothermia, there should not be any role or justification for use of low-cost cooling devices in LMICs [5,66,67].

#### 1.5.8 Magnetic resonance biomarkers in NE

Magnetic resonance (MR) biomarkers are useful in providing insights into the timing and nature of brain injury and in measuring treatment effects of neuroprotective interventions. These are particularly useful in early phase clinical trials as a ‘go’ or ‘no go’ surrogate endpoint, optimizing drug dosage and durations before phase III clinical trials, and in exploring sub-groups within a large clinical trial. MR spectroscopy biomarkers have the best prognostic accuracy, but until recently their use in multi-centre trials has been hampered by a lack of cross platform sequences to give comparable quantitively results from different scanner makes. As a part of the magnetic resonance imaging in NE (MARBLE) study, cross platform MS spectroscopy sequences were developed and validated in a large prospective cohort of 220 neonates with NE in the UK and the USA. The MARBLE study reported very high prognostic accuracy of MR spectroscopy, particularly thalamic N-acetyl aspartate absolute quantification [69].

The HELIX trial used the MARBLE sequences [69] and did not find any difference in the 3 T proton MR spectroscopy thalamic N-acetyl aspartate levels, brain injury scores on conventional MR imaging and whole brain fractional anisotropy between the hypothermic and usual care group, indicating lack of hypothermic neuroprotection [5]. On conventional MR scans, most neonates had white matter injury suggestive of partial prolonged hypoxia, and none of the neonates had evidence of established brain injury. One possible reason for lack of neuroprotection in the HELIX trial was that the secondary energy failure had already occurred by the time these neonates were born due to a partial prolonged intrapartum hypoxia. But these hypotheses need to be tested in future studies.

#### 1.5.9 Disease stratification in NE

The HELIX trial data highlights the heterogeneity of NE in LMICs and exposes substantial gaps in our understanding of the mechanisms of brain injury in these settings. Mechanistic studies and newer methods of classifying neonates with NE based on the underlying injury mechanism would be required to develop specific neuroprotective therapies. Specific gene expression signatures of neonates with NE have been described [70]. In a small sub-group of 45 neonates recruited to the HELIX trial, a total of 855 genes were significantly differentially expressed between the good and adverse outcome groups, of which RGS1 and SMC4 were the most significant [71]. Biological pathway analysis revealed over-representation of genes from pathways related to melatonin and polo-like kinase in neonate with adverse outcome [71]. Host transcriptomic profiling has the potential to make a paradigm shift in precision medicine for NE in LMICs settings.

#### 1.5.10 Specific neuroprotective therapies

Given the sub-acute nature of brain injury in LMICs [5], it is likely that drugs with neuroregenerative properties are more effective than acute neuroprotective therapies, until the underlying mechanisms are known. A recent systematic review of five small studies of erythropoietin monotherapy in LMICs, involving 348 neonates from tertiary neonatal intensive care units, showed a significant reduction in death or disability (RR 0.56; 95 % CI, 0.42–0.75), when administered within 24 h after birth [72]. Erythropoietin monotherapy needs to be evaluated in well designed and adequately powered randomized control trials in LMICs before clinical use, irrespective of the results of high-income country erythropoietin trials where erythropoietin is evaluated as an adjunct to cooling therapy, particularly as cerebral iron depletion may negate the effects of erythropoietin neuroprotection. The EMBRACE (Erythropoietin Monotherapy for Brain Regeneration in 10.13039/100006147NE) funded by Thrasher foundation recruiting over 800 neonates from South Asian neonatal intensive care units is expected to start recruitment soon.

Early phase clinical trials of stem cells are ongoing in HICs [73]. Given the unknown safety profile and potential adverse effects of stems cells, such therapies should establish their safety in phase III clinical trials in HICs before they are even considered for evaluation in LMICs. Further research into the nature and origins of brain injury in LMICs would be useful for developing appropriate neuroprotective strategies.

#### 1.5.11 Early developmental care

A subgroup of 164 neonates recruited to the ‘First Breath Study’ from India, Pakistan and Zambia who required resuscitation at birth were then randomized to an intensive home-based early developmental care program over three years or usual support only. Neonates with severe NE were excluded from randomisation, as early developmental care was expected not to improve outcomes in neonates with substantial brain injury. Only two neonates had moderate NE in this study and the remaining neonates had normal neurological assessments during neonatal period. Of these, 123 (75 %) of the neonates were assessed using Bayley II Scales of Infant Developmental at 3 years. Mental Developmental Index at 36 months was higher in the early developmental care group (102.6 ± 9.8) compared with the control group (98.0 ± 14.6, 1-sided, P = 0.02). Psychomotor Development Index was also higher in the early developmental care group (P = 0.04) [74]. Very few infants had disability at 36 months in both groups.

Several randomized controlled trials have assessed early developmental intervention for healthy neonates without substantial brain injury in LMICs [75]. In a single centre randomized controlled trial from south India that included 800 consecutive neonates admitted to special care baby unit, a low intensity, facility based early developmental intervention model of early stimulation therapy provided in the first year of the infants' lives led to a small but statistically significant difference in the Bayley Mental Development Index (MDI) at two years of age [76]. It is unclear if any of the recruited infants had NE, although 26 infants had Apgar scores between 4 and 6 at 1 min after birth. Again, very few infants had disability at 2 years of age in both groups, the logistics of undertaking over 700 Bayley examinations in these settings is not reported [76].

A recent cluster randomized control trial involving 1152 mother-child dyads reported a modest increase (approximately 5 Units) in Bayley cognitive scores with parent delivered early intervention program in Kenya in healthy infants [75]. Another meta-analysis of 13 studies (12 from HICs and 1 from a LMIC) analysing the impact of developmental care on the cognitive and motor outcomes in preterm neonates showed some benefit at 12 months; Mental development index (MDI) (standardized mean difference [SMD] 0.55; 95%CI, 0.23–0.87; p < 0.05), and psychomotor developmental index (PDI) (SMD 0.33; 95 % CI, 0.08–0.57; p < 0.05). However, there was no improvement in MDI at 24 months (SMD 0.15; 95 % CI, 0.05–0.35; p = 0.15), and the cost-benefits of intensive early developmental care remain unclear [77].

Future clinical trials are required to examine if early developmental care is beneficial for neonate with NE, and the data from healthy infants or infants born preterm should not be extrapolated to term neonates with brain injury.

#### 1.5.12 Ethical and governance issues in clinical trials in LMICs

There are several ethical and logistic challenges in undertaking NE research in LMICs, particularly amongst socio-economically disadvantaged populations. Parents may not understand the difference between research and clinical care and the concept of randomisation, nor may be empowered to refuse trial participation, even for experimental therapies with unfavorable risk benefit ratios.

A recent systematic review has reported parental consent rates of over 95 % for randomized controlled trial of neonatal interventions in LMICs, as opposed to less than 85 % in HICs, which raises serious concerns regarding research governance and parental understanding of the research process, particularly when the research is being conducted for commercial benefit by pharmaceutical companies [78].

In the HELIX trial, extensive training for the local team was conducted on research governance and informed consenting process, and the clinicians were instructed to avoid any coercion. Overall consent rate was 85 %. The consent process was video recorded and audited for quality assurance under three domains – empathy, information, and autonomy. Consistently high scores were obtained under these domains indicating adequate quality of informed consent, i.e., appropriate information was provided to parents. Subsequently, qualitative interviews with parents and professional were conducted to explore their views about the research participation [51]. Thematic analysis of the data suggested that the parental decision to participate was primarily based on an unreserved trust in the treating doctors, therapeutic misconception, and the opportunity to have an expensive treatment free of cost. Most parents did not understand the concept of a clinical trial nor the nature of the intervention, despite detailed explanations about the study. Interviews with professionals (prior to the data analysis of the HELIX trial) showed a strong bias towards cooling therapy, as this was already a standard therapy in HICs and in most hospitals in LMICs [51].

The HELIX trial was conducted in settings with relatively high literacy (over 90 %) rates in South India [5]. If these parents are unable to grasp the concept of research in such settings, it is very unlikely that parents in sub–Saharan African settings with much lower literacy rates can comprehend the complexities of neonatal research, particularly in drug trials where the clinical teams or investigators have a vested interest. Hence, further work on parent and community engagement and involvement and health literacy is vital in research in LMICs and written informed parental consent alone may have little meaning. The researchers should be encouraged to monitor the consenting process and parental engagement by audits of audio-visual recording and to undertake and report parental understanding of the trial participation using nested qualitative studies in randomized controlled trials. Medical journals should ensure this process as a part of the consort guidelines.

### 1.6 Future directions

The randomized controlled trials in HICs are almost always conducted by experienced researchers and supported by clinical trials units with strong research governance systems to ensure the data integrity. In contrast, clinicians in LMICs rarely have protected research time, nor have adequate access to high-quality and affordable perinatal trials unit. Hence, clinical trials are often conducted by a trainee doctor with no research experience, while undertaking full time clinical work.

While international collaborations bring in considerable valuable and credibility in NE research in LMICs, it is essential that the research is conducted in an ethical way and entirely based on the needs of the local populations, and not the ambitions of the HICs alone. For example, it would be inappropriate to conduct a cooling trial or early phase neuroprotective drug trial in a sub-Saharan neonatal unit that lacks basic neonatal care; rather, the priority should be to improve basic neonatal and antenatal care.

In contrast, settings with good facilities for antenatal and neonatal intensive care could focus on neonatal neuroprotection trials in addition to primary prevention of NE. Specific neuroprotective therapies should be evaluated in settings with good supportive neonatal intensive care facilities before they are tested in those without such facilities.

International funding bodies have a responsibility to ensure the needs of the population in LMICs are the primary focus of research, particularly when high income country researchers are undertaking research in LMICs. The National Institute for Health Research (NIHR) in the UK have set benchmarks for equitable collaborations between partners in HICs and LMICs, with academic research capacity building and community engagement and involvement at the center of any research collaboration following the ESSENCE principles [79]. ‘Parachute research’ by researchers in HICS in LMICs is strongly discouraged [80]. It should no longer be acceptable for all co-authors in a trial in LMICs [50] to be from HICs, and only token authorship is given to the local investigators who undertake the work [81]. Given the heterogeneity of NE in LMICs and negative results of the HELIX trial [5] despite very strong pre-clinical data on hypothermic neuroprotection [82], it is unlikely that data from animal models can inform neuroprotection in these settings. Although double-hit pre-clinical models of post-natal sepsis and ischemia has been developed [83], these are not representative of NE in LMICs [5], and may provide misleading information.

Parents and community should not be seen as mere participants in research nor as ceremonial members in research proposals. They should be actively involved in all aspects of the work including identification of the research needs. Hence, prior training of both parents and the wider community workers are required so that they are able to provide meaningful contributions to research. Academic capacity building in LMICs should be central in any collaborative projects between HICs and LMICs, and funding bodies have a responsibility to ensure this. Multi-disciplinary collaboration between obstetricians, public health experts, neonatologists, neurologists, social scientists, and other stakeholders are essential to develop holistic programs of research for preventing and managing NE in LMICs. A Global South Preventable Neurodevelopmental Disability Alliance (http://neurodisabilitiesalliance.com) has been recently established to undertake high quality research into NE in LMICs.

## 2 Conclusion

Despite high levels of facility-based deliveries, the incidence of NE remains high, leading to a substantial burden of preventable childhood neurodisabilities like cerebral palsy, epilepsy, deafness, and blindness in LMICs. Limited high qualitive evidence is available on prevention and management of NE in settings. Therapeutic hypothermia despite optimal neonatal intensive is ineffective and harmful in South Asian tertiary neonatal intensive care units, highlighting the dangers of extrapolating evidence from HICs to LMICs without adequate evaluation in rigorous randomized controlled trials. Maintaining normothermia at all times, avoidance of hyperoxia, hypocarbia, hypoglycemia, and good supportive care, including cardiovascular and ventilatory support, may help improve outcomes. Future studies should explore the origins and timing of birth injuries, effective intrapartum monitoring, supportive neonatal care, disease stratification based on underlying mechanisms and precision medicine for neuroprotection and neuro regeneration. Equitable partnerships and academic capacity building in LMICs should be central to future research in these settings.

## Contributions

VKr and VKu drafted the first draft of the manuscript under the supervision of ST. All authors contributed to the further development of the manuscript and approved the final version. ST is the guarantor of the work.

## Funding

VKr and VKu are funded by an NIHR Research and Innovation for Global Health Transformation (RIGHT) program grant, and ST is supported by an NIHR advanced fellowship. The views expressed are those of the author(s) and not necessarily those of the NIHR or the Department of Health, UK.

## Practice points

• Therapeutic hypothermia alongside optimal supportive care increases mortality and does not provide neuroprotection after NE in tertiary neonatal intensive care units in south Asia.
• Meticulous attention to supportive care, including avoidance of hyperthermia, hyperoxia, hypocarbia, hypoglycemia, and appropriate seizure management, remain the most important steps to reduce brain injury.

## Research directions

• Research in LMICs should be based on the needs of the local population with a strong focus on equitable partnerships with HICs partners and local capacity building.
• Empowering parents and families, exploring novel ways of research consenting and nested qualitative studies to explore parental understanding of research participation is important to ensure research in LMICs adheres to the highest ethical standards.
• In low resourced sub-Saharan African settings with high rates of home births, focus should be on increasing hospital-based births and in providing basic neonatal care, rather than on advanced neuroprotection research.
• In settings with high facility-based births, mechanistic, advanced neuroimaging and neurophysiological studies may help to understand the nature, origins and timing of brain injury in NE, and in developing precision medicine for appropriate neuroprotective therapies.
• In neonatal intensive care units with facilities for invasive ventilation and cardio-vascular monitoring, randomized control trials to develop evidence-based interventions for supportive care including thresholds for blood product replacements, glycemic control, correction of metabolic acidosis, neonatal seizures, and neuro-regenerative therapies should be conducted.
